# Differential Symptomology, Susceptibility, and Titer Dynamics Manifested by Phytoplasma-Infected Periwinkle and Tomato Plants

**DOI:** 10.3390/plants13060787

**Published:** 2024-03-10

**Authors:** Algirdas Ivanauskas, Junichi Inaba, Yan Zhao, Kristi D. Bottner-Parker, Wei Wei

**Affiliations:** 1 Molecular Plant Pathology Laboratory, Beltsville Agricultural Research Center, Agricultural Research Service, United States Department of Agriculture, Beltsville, MD 20705, USA; algirdas.ivanauskas@usda.gov (A.I.); junichi.inaba@usda.gov (J.I.); yan.zhao@usda.gov (Y.Z.); kristi.bottner@usda.gov (K.D.B.-P.); 2 Laboratory of Plant Pathology, Nature Research Centre, Akademijos Str. 2, LT-08412 Vilnius, Lithuania

**Keywords:** phytoplasma, virescence, phyllody, witches’-broom, big bud, cauliflower-like inflorescence, meristem, susceptibility, intracellular bacteria

## Abstract

Phytoplasmas are intracellular pathogenic bacteria that infect a wide range of plant species, including agriculturally important crops and ornamental trees. However, our understanding of the relationship between symptom severity, disease progression, and phytoplasma concentration remains limited due to the inability to inoculate phytoplasmas mechanically into new plant hosts. The present study investigated phytoplasma titer dynamics and symptom development in periwinkle and tomato, both infected with the same potato purple top (PPT) phytoplasma strain using a small seedling grafting approach. Virescence, phyllody, and witches’-broom (WB) symptoms sequentially developed in periwinkle, while in tomato plants, big bud (BB, a form of phyllody), cauliflower-like inflorescence (CLI), and WB appeared in order. Results from quantitative polymerase chain reaction (qPCR) targeting the PPT phytoplasma’s 16S rRNA gene revealed that in both host species, phytoplasma titers differed significantly at different infection stages. Notably, the highest phytoplasma concentration in periwinkles was observed in samples displaying phyllody symptoms, whereas in tomatoes, the titer peaked at the BB stage. Western blot analysis, utilizing an antibody specific to PPT phytoplasma, confirmed substantial phytoplasma presence in samples displaying phyllody and BB symptoms, consistent with the qPCR results. These findings challenge the conventional understanding that phytoplasma infection dynamics result in a higher titer at later stages, such as WB (excessive vegetative growth), rather than in the early stage, such as phyllody (abnormal reproductive growth). Furthermore, the PPT phytoplasma titer was markedly higher in periwinkles than in tomato plants, indicating differing susceptibilities between the hosts. This study reveals distinct host responses to PPT phytoplasma infection, providing valuable insights into phytoplasma titer dynamics and symptom development, with implications for the future management of agricultural disease.

## 1. Introduction

Phytoplasmas are a group of phloem-restricted bacteria that cause various diseases across a wide range of plant species, significantly impacting agriculture and horticulture worldwide [1,2]. These bacteria possess small genomes, ranging from 530 to 1200 kb, which reflect their intracellular and parasitic lifestyle, as they depend heavily on host plants for nutrients [3,4,5]. Similar to other intracellular pathogens [6,7], phytoplasmas can exploit host cells for survival and multiplication while evading the host’s immune system [8]. Once phytoplasmas encounter a host plant and start to establish infection, the plant recognizes the pathogen and activates defense mechanisms. When the invading phytoplasma overcomes/evades the host’s defenses, the plant becomes susceptible to infection, developing disease symptoms. Typical symptoms include yellowing of leaves, stunting, virescence (the greening of non-green plant flower parts, VIR), phyllody (where flowers transform into leaf-like structures, PHY), and witches-broom (WB) [9,10,11,12,13].

Phytoplasmas lack a cell wall and cannot be cultured in artificial media [2]. Unlike many plant-pathogenic bacteria and viruses, which can often be mechanically inoculated to new plant hosts for research purposes, phytoplasmas cannot be similarly manipulated in the laboratory setting [14]. Instead, the inoculation/transmission of phytoplasmas from one plant to another primarily relies on insect vectors, grafting techniques, and the use of parasitic plants like dodder [13,15,16]. However, these inoculation methods often lack the precision required to effectively control the amount of the inoculum and observe early host response events. This hinders the elucidation of the exact relationship between phytoplasma titer and symptom development within a single host plant.

Despite these challenges, a few studies with well-designed inoculation approaches have indicated that phytoplasmas, akin to plant viruses, can establish themselves at the site of inoculation and then spread systemically throughout the host plant. Invading phytoplasmas typically migrate first to the plant’s sink tissues, such as the apical stem, newly expanded leaves, and roots, and then advance to the source tissues, such as mature leaves. This movement pattern has been observed in cases such as the onion yellows phytoplasma in garland chrysanthemum using localized inoculation of insect vectors [14] and ‘*Candidatus* Phytoplasma solani’ in MicroTom tomatoes by employing graft inoculation [17]. As phytoplasmas reach and colonize the target site, their concentration, or titer, tends to increase, facilitating further spread and colonization within the plant [14,17]. However, the correlation between phytoplasma concentration changes and progressive symptom development remains understudied.

To address this knowledge gap, the present study employed a small seedling grafting technique that was previously developed [12]. This approach enables the observation of sequentially developed symptoms in tomato (Moneymaker) plants infected with potato purple top (PPT) phytoplasma. Typically, symptoms such as big bud (BB), cauliflower-like inflorescence (CLI), and witches’-broom (WB) emerged at around 28-, 45- and 60-days post inoculation (dpi) [12]. In the present study, the investigation was extended to explore periwinkles infected with the same PPT phytoplasma strain, where a sequential appearance of symptoms, including VIR, PHY, and WB, was observed. By conducting qPCR, the changes in the titer of PPT phytoplasma within periwinkle and tomato plants were examined throughout the development of disease symptoms.

Our initial hypothesis assumed that the titer of PPT phytoplasma would exhibit a general trend during the course of infection progression. Specifically, we expected the phytoplasma titer to be low during the early stages of infection, characterized by symptoms such as VIR and PHY, and to subsequently increase as the infection progressed into more advanced stages, like WB. However, the qPCR results unveiled a different and interesting pattern in the dynamics of PPT phytoplasma titer within the infected plants. Contrary to conventional expectations, the relatively higher phytoplasma titer was not observed during WB (50/60 dpi in periwinkle and tomato, respectively) but rather at the earlier BB/PHY infection stage (35/28 dpi in the same two hosts). These unexpected observations suggest a dynamic relationship between phytoplasma titer and symptom development, which could have important implications for future research.

In addition, the present study also revealed that PPT phytoplasma titer was significantly higher in periwinkles than in tomato plants. This strongly indicates varied susceptibility of periwinkle and tomato to PPT phytoplasma infection. This study sheds light on the complex interplay between phytoplasma infection dynamics, symptomatology, and differential host specificity concerning susceptibility and resistance, offering valuable insights into disease management and crop protection strategies.

## 2. Results

### 2.1. PPT Phytoplasma-Induced Symptoms in Tomato and Periwinkle Plants

**Figure 1:**
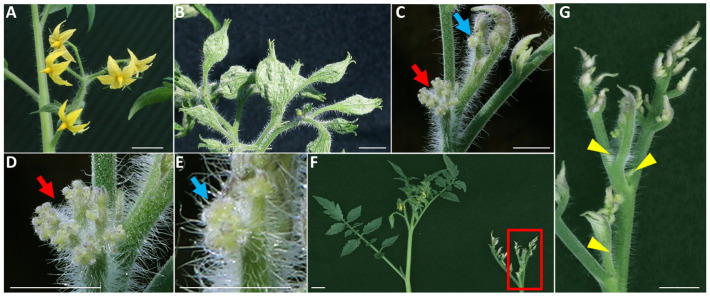
Potato purple top (PPT) phytoplasma-induced symptoms in tomato (Moneymaker) plants. (**A**), A mock-inoculated inflorescence bearing flowers. (**B**,**C**), PPT phytoplasma infection caused big bud (BB) and cauliflower-like inflorescence (CLI). The red and blue arrows indicate the CLI structures, and closeup images of CLI are shown in (**D**,**E**), respectively. (**F**), A mock-inoculated plant with leaves and inflorescence (left panel), and PPT phytoplasma infection-induced witches’-broom (WB, right panel). (**G**), Closeup image of the red box of (**F**); Yellow triangles point to the lateral buds initiated from the leaf axils. Scale bar = 1 cm.

**Figure 2:**
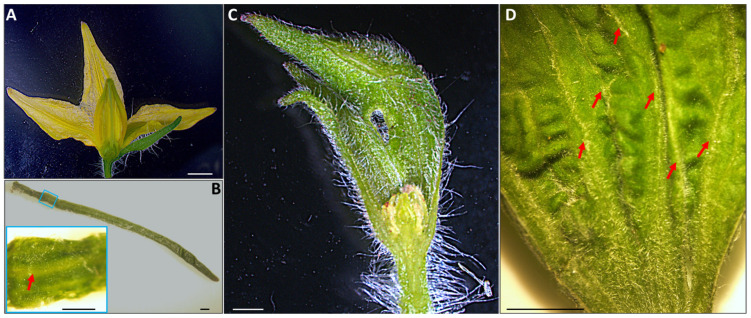
Stereomicroscopic observation of potato purple top (PPT) phytoplasma-induced big bud (BB) symptom in tomato (Moneymaker) plants. (**A**), A dissected mock-inoculated tomato flower, including sepals, petals, stamens, and carpel. (**B**), A sepal collected from a mock-inoculated tomato flower. A close-up image of a partial sepal is provided in the blue box in the left corner, with a red arrow indicating a leaf vein-like structure. (**C**), A dissected enlarged and fused sepals from a BB, where inner flower organs (petals, stamens, and carpel) were underdeveloped. (**D**), Additional leaf vein structures were observed in the sepals derived from BB. Scale bar = 0.5 cm.

**Figure 3:**
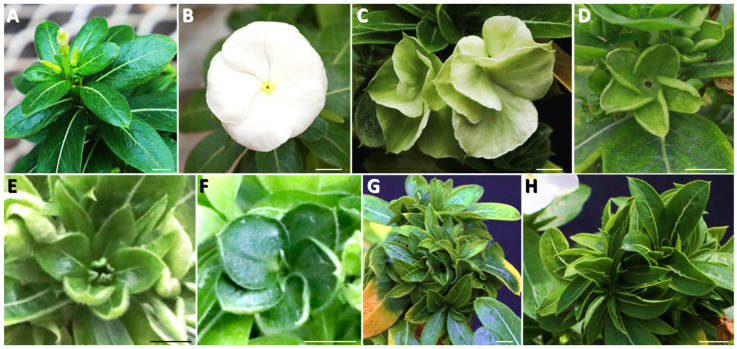
Potato purple top (PPT) phytoplasma-induced symptoms in periwinkle (Vinca Pacifica XP) plants. (**A**,**B**) A mock-inoculated periwinkle plants with leaves (**A**) and solitary flower (**B**). (**C**–**H**) PPT phytoplasma infection caused virescence (VIR), phyllody (PHY), and witches’-broom (WB) symptoms, respectively. Scale bar = 1 cm.

This study investigated the dynamic variations in PPT phytoplasma concentrations within two plant hosts, tomato, and periwinkle. The tomato variety Moneymaker and periwinkle (Vinca Pacifica XP pure white) were employed. Both plants show indeterminate growth, characterized by continuous development throughout the plant life cycle, allowing it to add new tissues and structures as long as it remains healthy, and conditions are favorable. These two host plants exhibited varying symptoms despite being infected with the same PPT phytoplasma strain (Figure 1, Figure 2 and Figure 3).

In the case of PPT phytoplasma-infected tomato plants, BB, CLI, and WB symptoms (Figure 1B–G) sequentially developed at approximately 28-, 45-, and 60- days post-graft inoculation (dpi). Each symptom corresponds to a distinct stage of phytoplasma infection/disease progression [12]. Compared to mock-inoculated flowers (Figure 2A,B), BB exhibits the enlargement of the fused sepals (the outermost protective floral parts) and a reduction in the inner three floral organs, typically petals, and reproductive structures like stamens and carpels (Figure 1B and Figure 2C). BB is a form of phyllody characterized by the flower organs transforming into leaf-like structures (more leaf veins, Figure 2D). This is caused by the premature termination of the floral meristem, which has a terminal fate [12]. Furthermore, a comparison was conducted between the sepals collected from mock-inoculated flowers and those obtained from infected big buds (BB). The enlarged and fused sepals from the single BB showed significant morphological differences compared to the mock sepals per flower. Specifically, they were found to be 2.98 times longer and 4.46 times wider than their mock counterparts. The infected sepals exhibited a remarkable 9.86-fold increase in weight compared to the mock sepals (Table 1).

CLI was characterized by its unique structure resembling cauliflower (Figure 1C–E). This symptom results from the formation of repetitive inflorescence meristems without initiating further floral meristems [12]. WB is identified by the clusters of small, tightly packed shoots or branches that emerge from every leaf axil, often resembling a broom (Figure 1F,G; [12]). Our earlier study has revealed that when phytoplasma infects a plant, it disrupts sugar metabolism and impedes sucrose transportation. The sucrose was reallocated to the leaf axil, triggering the initiation of axillary buds. This process is accompanied by the distribution of large amounts of cytokinin at the leaf axil. Repeated initiation and outgrowth of axillary buds and leaf premature senescence eventually manifest as WB symptom [18]. BB was observed during the early stage of infection, on the first inflorescence, and occasionally persisted until the second inflorescence. However, as the infection advanced, the subsequent inflorescences displayed CLI (without visible flower organs due to arrested floral meristems); No new BB would form at this stage. Consequently, the infected plants underwent a transition toward vegetative growth, characterized by the WB appearance with more leaves and CLI inflorescences.

A periwinkle plant typically enters the flowering phase after growing 6 to 8 sets of true leaves. During this phase, the periwinkle plant’s bloom pattern becomes visible, with the emergence of individual flowers at each leaf axil (Figure 3A,B) [19,20]. In PPT phytoplasma-infected periwinkle plants, a sequential progression of symptoms that included VIR (Figure 3C,D), PHY (Figure 3E,F), and WB symptoms (Figure 3G,H) were observed. These symptoms manifested at distinct intervals, occurring at approximately 30-, 35-, and 50 dpi. Both VIR and PHY affected the petals of infected periwinkle flowers, changing from their original colors to green with a leaf texture during the VIR stage and then evolving into full leaf-like structures at the PHY stage. The transition from VIR to PHY stages occurred relatively quickly, with a narrow window for observation. Similar to the infected tomato plants, the symptoms of VIR and PHY were detectable only in the early stages of infection, followed by a shift in infected plants towards WB, a more vegetative growth pattern (Figure 3G,H).

To capture the temporal dynamics of phytoplasma proliferation and symptom development in periwinkle, samples from infected plants at different symptom stages, including VIR, PHY, and WB (30, 35, and 50 dpi), and samples from tomato at various symptom stages, including BB, CLI, and WB (28, 45, and 60 dpi), were examined and compared.

### 2.2. Standard Curves

To quantify phytoplasma titers, qPCR was performed on the DNA samples from infected plants to target the 16S rRNA gene, a well-established genetic marker within the phytoplasma genome [21]. The plant DNA abundance in the DNA samples was also assessed using the 18S rRNA gene for tomato and the actin gene for periwinkle. The ratio of phytoplasma titer to plant DNA abundance can also serve as a valuable marker for quantifying unculturable phytoplasmas in plant systems. This approach has been widely reported [22,23]. Both quantification of the PPT-16S rRNA gene alone and comparison of the ratio of PPT and plant DNA were adopted in this study.

**Figure 4:**
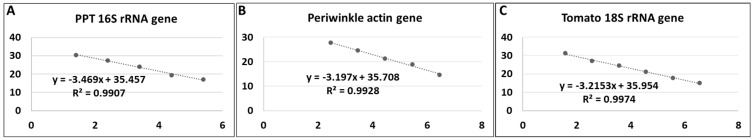
Standard curves for the quantitative PCR (qPCR) analysis of three genetic markers: (**A**), the 16S rRNA gene of the Potato Purple Top (PPT) phytoplasma. (**B**), the actin gene of periwinkle, and (**C**), the 18S rRNA gene of tomato. These curves were generated by plotting the cycle threshold (Ct) values (*y*-axis) against the logarithm (base 10) of known concentrations of plasmid DNA (*x*-axis) containing the respective target genes.

The 16S rRNA gene of the PPT phytoplasma, the 18S rRNA gene of tomato, and the actin gene of periwinkle were amplified by PCR using specific primers, P1/16S-SR and P1A/16S-SR [24,25], Tom18SbF1/Tom18SbR1, and CrAct3bF1/CrAct3bR1, respectively (Table 2). The generated PCR amplicons of three genes varied in length: 1530 bp, 1144 bp, and 945 bp. These amplicons were cloned into the TOPO cloning vector, and the concentration of each plasmid DNA containing a specific gene fragment was measured, ranging from 200–300 ng/µL. Subsequently, these tenfold serially diluted plasmid DNA samples (10^−4^ to 10^−9^) were subjected to qPCR. Through qPCR analysis, the standard curves of three targeted genes were constructed by establishing a correlation between the cycle threshold (Ct) values obtained in our experiments and the known log10 concentrations of plasmid DNA containing the respective genes, as shown in Figure 4 (Figure 4A–C). The R^2^ values of standard curves derived from 16S rRNA, 18S rRNA, and actin genes were 0.9907, 0.9928, and 0.9974, nearing the perfect score of 1.00. These results suggest the high efficiency and reliability of the qPCR reactions for accurately quantifying these target genes. These standard curves facilitated the quantification of phytoplasma and enabled an indirect assessment of plant DNA content in our experimental samples.

### 2.3. PPT Phytoplasma Concentration in Host Plants

DNA extraction from host plants was carried out at various infection stages, specifically VIR, PHY, and WB stages in periwinkle, and BB, CLI, and WB stages in tomato, followed by qPCR analysis. The primer set MPPLPPT16SF2/MPPLPPT16SR2 was employed to quantify the 16S rRNA gene of PPT phytoplasma [21]. The Tom18SinF1/Tom18SbR1 and CrAct3inF2/CrAct3bR2 primers (Table 2) were used to measure the 18S rRNA gene of tomato and the actin gene of periwinkle, respectively.

**Figure 5:**
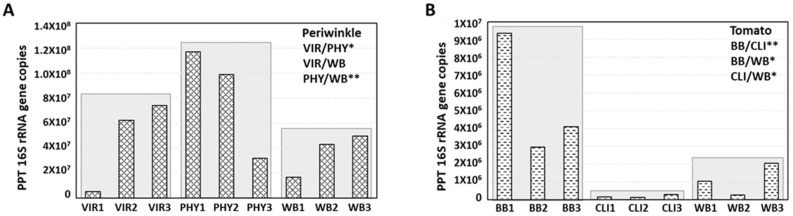
Quantitative PCR (qPCR) analysis-based measurement of the copy numbers of the 16S rRNA gene as an indicator of potato purple top (PPT) phytoplasma populations in infected periwinkle and tomato plants. The analysis included (**A**) periwinkle samples exhibiting symptoms of virescence (VIR), phyllody (PHY), and witches’-broom (WB) symptoms, as well as (**B**) tomato samples with big bud (BB), cauliflower-like inflorescence (CLI), and WB symptoms. This allowed for the assessment of phytoplasma abundance corresponding to specific disease manifestations in these plant hosts. Asterisk (*) and double asterisk (**) denote statistical significance (*p* < 0.05 and *p* < 0.01, respectively) as determined by the Student’s *t*-test.

In PPT phytoplasma-infected periwinkle plants, the 16S rRNA gene copies (per microliter sample DNA) ranged from 5.33 × 10^6^ to 7.43 × 10^7^, 3.17 × 10^7^ to 1.17 × 10^8^, and 1.68 × 10^7^ to 4.96 × 10^7^ at the VIR, PHY, and WB infection stages, respectively (Figure 5A). These findings showed an increase in phytoplasma concentration from the VIR to the PHY stage. Such titer increase aligned well with the gradual morphological changes that occurred in virescent flowers and phyllody flowers, where infected petals exhibited a transition from a pale green color with some leaf-like texture at the VIR stage to a full leaf-like structure at the PHY stage (Figure 3C–F). The latter structure possesses well-developed phloem tissues suitable for phytoplasma colonization. There was no significant difference in titer between VIR and WB samples, indicating that the highest PPT phytoplasma titer was in the DNA samples collected from flowers displaying phyllody symptoms. Similarly, in tomato plants infected with PPT phytoplasma, the 16S rRNA gene copy number was highest during the BB infection stage, ranging from 2.96 × 10^6^ to 9.35 × 10^6^/µL (Figure 5B). In addition, the lowest PPT phytoplasma titer was observed during the CLI stage (Figure 5B). This is expected and can be attributed to the composition of the CLI structure, which consists of repetitively initiated inflorescence meristems where phytoplasmas cannot reach and colonize [12]. Additionally, in both periwinkle and tomato plants, phytoplasma-induced WB symptoms developed relatively later than VIR, PHY, BB, and CLI. However, there was no observable upward trend in phytoplasma titer at the WB stage after the BB/PHY stage (Figure 5A,B).

**Figure 6:**
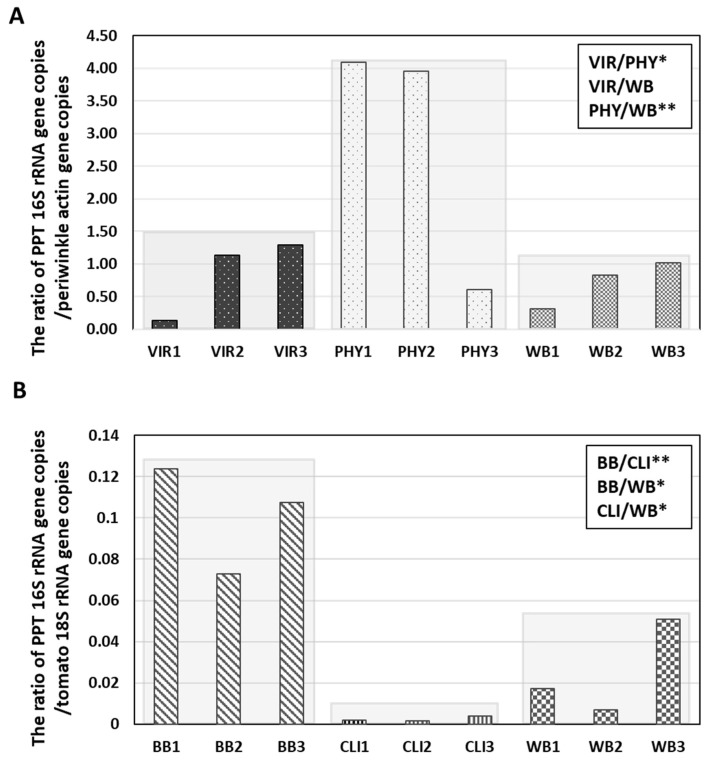
The relative abundance of potato purple top (PPT) populations in the infected host plants, including (**A**) periwinkle and (**B**) tomato. (**A**) the ratio of PPT phytoplasma 16S rRNA gene copies to actin gene copies of periwinkle, exhibiting symptoms of virescence (VIR), phyllody (PHY), and witches’-broom (WB) symptoms. (**B**) the ratio of PPT phytoplasma 16S rRNA gene copies to 18S rRNA gene copies of tomato with big bud (BB), cauliflower-like inflorescence (CLI), and WB symptoms. Asterisk (*) and double asterisk (**) denote statistical significance (*p* < 0.05 and *p* < 0.01, respectively) as determined by the Student’s *t*-test.

In addition to assessing the PPT phytoplasma titer through the quantification of the 16S rRNA gene copy number, the relative ratio of phytoplasma DNA to plant DNA was also calculated as an additional indicator to provide further insights into the abundance of the phytoplasma population. Consistent with the results obtained from the measurement of PPT-16S rRNA gene copies, the ratio of PPT phytoplasma to periwinkle DNA (expressed as 16S rRNA to actin gene) exhibited a range, spanning from 0.61 to 4.09 in the PHY samples. This range was the highest among all DNA samples obtained from periwinkle (Figure 6A). In contrast, within tomato samples, the PPT/tomato DNA ratio (expressed as 16S rRNA to 18S rRNA gene) peaked in BB samples, ranging from 0.07 to 0.12 (Figure 6B). These observations collectively suggest that the PPT phytoplasma is more abundant in the PHY and BB samples, representing the early infection stages in the plant.

Furthermore, a clear pattern emerges when considering the direct measurement of PPT titer and the relative ratio of PPT versus plant DNA. The PPT phytoplasma population appears significantly more abundant in periwinkle than in tomato plants (Figure 5 and Figure 6). This finding highlights the distinct susceptibility of periwinkle and tomato to PPT phytoplasma infection, with periwinkle harboring a higher pathogen load.

### 2.4. PPT Phytoplasma Titer Indicated by IDP Level

To further assess the phytoplasma abundance, western blot analysis was employed as a double-blind confirmation method by using a polyclonal antibody, referred to as the anti-PPT-IDP antibody, which was generated in our previous study [8]. This antibody is specific to PPT phytoplasma’s immunodominant membrane protein (IDP). IDP occupies a substantial portion of the phytoplasma cellular membrane, enabling visualization/detection of phytoplasma within infected plant samples, as documented in previous studies [8,14]. Western blot analysis with the anti-PPT-IDP antibody indirectly assessed the presence and the relative abundance of PPT phytoplasma in the infected plants.

**Figure 7:**
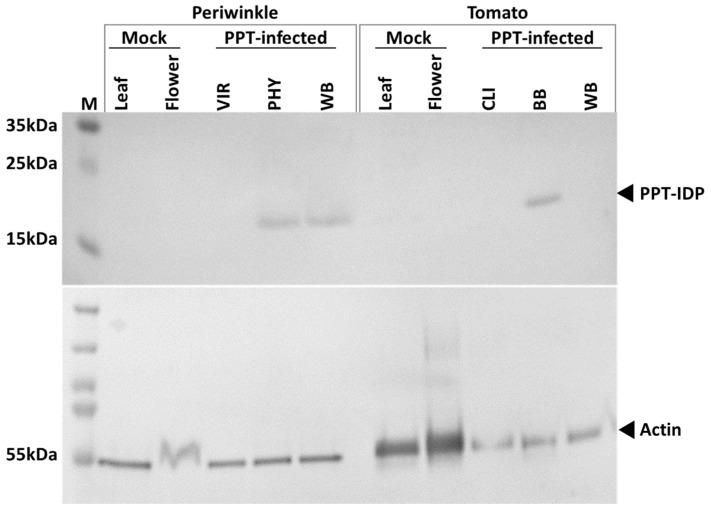
Measurement of potato purple top (PPT) phytoplasma abundance using western blot analysis. A polyclonal antibody targeting immunodominant membrane protein (IDP) of PPT phytoplasma was employed to measure PPT-IDP expression levels. These levels serve as an indirect indicator of phytoplasma abundance in infected plants. Especially the periwinkle samples manifesting virescence (VIR), phyllody (PHY), and witches’-broom (WB) symptoms, along with tomato samples presenting with big bud (BB), cauliflower-like inflorescence (CLI), and WB symptoms. An actin antibody was employed as the loading control. Marker (M): PageRuler™ Plus Prestained Protein Ladder, ranging from 10 to 250 kDa.

Total protein extractions were performed on both PPT phytoplasma-infected periwinkle and tomato plants, utilizing the identical infection stages and sample sources as those employed for our qPCR quantification. In periwinkle, the distinct 16.9 KDa bands specific to the IDP protein were observed in both PHY and WB samples (Figure 7), indicating the presence of PPT-IDP and hance PPT phytoplasma. PPT-IDP was also detected in the BB samples collected from infected tomato plants (Figure 7). No PPT-IDP was detected in the VIR samples from periwinkle and the CLI and WB samples from tomato. This may be attributed to the relatively low sensitivity of western blot analysis, along with the different composition and characteristics of plant tissues (Figure 7). These results indicate that phytoplasma abundance is higher in samples, including BB from tomato plants and PHY and WB in periwinkle.

In addition, actin was used as a reference control protein. The intensity of the IDP and actin bands was quantified using the histogram function in Adobe Photoshop, where white corresponds to RGB 255,255,255, and black to RGB 0,0,0. Lower histogram values represent higher intensity. Upon analysis, the intensity ratio of PPT-IDP to actin was 2.09 (121/58) in PHY samples and 2.64 (124/47) in WB samples obtained from periwinkle. This result suggests a slightly higher PPT-IDP intensity in PHY samples than in WB samples. In conclusion, our results revealed that IDP expression, which reflects the phytoplasma abundance, is most pronounced in PHY and BB samples. This finding was consistent with the qPCR results, reinforcing the accuracy and reliability of the concentration assessment results.

## 3. Discussion

Understanding the relationship between pathogen concentration and plant disease progression is crucial for effectively diagnosing, managing, and controlling these diseases. Such a relationship provides insights into the severity of the infection, the stage of the disease, and the plant’s response. In many plant diseases, there is a notable correlation between the severity of disease symptoms and the increase in pathogen titer within the infected plant tissues. For example, in viral diseases such as tomato yellow leaf curl disease (TYLCD), the most severe symptoms typically correspond with the highest concentration of virus particles in the leaves [26]. Similarly, in citrus trees infected with *Spiroplasma citri*, the severity of disease symptoms is correlated with the pathogen load [27]. These examples demonstrate a common trend where peak symptom severity coincides with the highest pathogen titer.

In the context of phytoplasma infection, it is well-documented that phytoplasma induces floral reversion, leading to symptoms like virescence and phyllody (Figure 1E–G, [11,12,13,28]). Additionally, there is an increase in leaf production, which results in a decrease in reproductive growth and an extension of vegetative growth [12]. It is generally assumed that the titer of phytoplasma would be higher in vegetative tissues than in deformed reproductive tissues. Several reports also revealed the highest titer in the infected leaves (especially source leaves), stems, and roots [29,30,31]. Furthermore, previous studies have shown that phytoplasma titers tend to increase in various tissue types over time following inoculation by insect vectors or through grafting, particularly in the early stages of infection [14,17]. Factors such as the concentration of phytoplasmas in the inoculum (for example, graft scions) and the timing of their introduction into the plants are crucial in determining the onset and development of symptoms, as described in our previous study [32]. However, the precise relationship between the phytoplasma titer within the plant and the progression of symptoms remains elusive.

To better understand these dynamics, the present study investigated phytoplasma colonization and symptom development in two host plants, including periwinkle and tomato. In periwinkle, symptoms such as VIR, PHY, and WB developed sequentially post-infection (Figure 3). BB, CLI, and WB appeared at different stages post-infection in tomato plants (Figure 1; [12]). VIR was absent in tomato plants, and CLI was not observed in periwinkle, reflecting distinct growth habits and patterns of different plant hosts, along with their corresponding responses. Most importantly, symptoms such as BB, VIR, and PHY, which manifest as flower organ deformation, are transitionary and only observable during the early stages of infection.

The qPCR analysis revealed apparent variations in PPT phytoplasma titer at different infection stages in both periwinkle and tomato. Specifically, in periwinkle, the highest phytoplasma titer was observed in DNA samples extracted from phyllody flowers (Figure 5 and Figure 6). On the other hand, tomato plants exhibited their highest phytoplasma titer during the BB (a form of phyllody) infection stage. This finding suggests a strong association between high phytoplasma titer and phyllody symptoms in floral organs (Figure 5 and Figure 6). To further confirm these observations, western blot analysis using the anti-PPT-IDP antibody was used to serve as an additional means to indirectly assess the presence and titer of phytoplasma in the infected plants. The results closely aligned with the qPCR findings, with the expression of IDP, indicative of the phytoplasma concentration, being most pronounced in samples exhibiting phyllody symptoms, specifically PHY and BB, in both periwinkle and tomato plants (Figure 7). The studies on phytoplasma titer in infected flowers are limited, with only a few documented cases [31,33,34]. For instance, in experimental periwinkle plants infected with peanut witches’-broom phytoplasma, the deformed flowers show a relatively high titer of phytoplasma [33]. In almonds, a relatively lower concentration of almond witches’ broom phytoplasma was observed in infected petals [31].

Contrary to the conventional belief that elevated phytoplasma titers would be found in tissues displaying late-stage symptoms like WB, which shift towards excessive vegetative growth, this study revealed that higher concentrations were present earlier in the reproductive tissues, manifested by phyllody symptoms. These results point to a more intricate and dynamic interaction between phytoplasma infection and plant symptom development, suggesting that the processes governing pathogen proliferation and symptom manifestation are more complex than previously thought. The early increase in phytoplasma titer during the phyllody stage might be related to the plant’s immune response. As the plant recognizes and attempts to defend against the pathogen, the phytoplasmas may counter by rapidly replicating, leading to the observed higher titer at this stage. Further studies are needed to understand the molecular mechanisms underlying the complex interplay between phytoplasmas and plant immunity.

PHY/BB, which involves the transformation of floral parts into leaf-like structures, results from the premature termination of the floral meristem induced by phytoplasmas [12,13]. This implies that phytoplasmas may actively manipulate host plant physiology early in the infection to create a more conducive environment for their proliferation. Phytoplasma-induced phyllody may be beneficial to the phytoplasma. These leaf-like structures have more abundant and accessible phloem compared to regular floral tissues (Table 1; Figure 2C,D), providing an enhanced environment for the phytoplasmas to thrive and multiply. This may also account for the higher abundance of phytoplasma observed in PHY/BB samples.

WB is characterized by excessive shoot branching, which occurs in the upper regions of infected plants. A previously established model [18] has revealed that phytoplasma infection disrupts sugar metabolism and obstructs sucrose transportation through the phloem. Consequently, sucrose is reallocated to the leaf axils, triggering the initiation and outgrowth of excessive lateral buds. The newly developed leaves and shoots from the lateral buds were compact and smaller in size, resembling the appearance of a broom. A separate study has shown that these small leaves contain fewer phloem tissues compared to mock controls (unpublished data). This disparity in phloem tissues may have contributed to the relatively low phytoplasma titer observed in leaves exhibiting WB symptoms.

From the plant’s perspective, floral reversion (phyllody and virescence) and WB (increased number of leaves) due to phytoplasma infection represent a trade-off. This trade-off balances the benefits of enhanced vegetative growth and photosynthetic capacity against the cost of compromised reproductive capabilities. This physiological shift could boost the plant’s energy production and might assist in coping with stress. Our preliminary study demonstrated that the fused and enlarged sepals collected from a single big bud exhibit higher levels of chlorophyll and elevated sucrose accumulation than sepals of a single flower at the equivalent stage in the mock plant (unpublished data). These observations strongly imply that infected plants have an increased demand for nutrients and energy support in response to phytoplasma infection.

Moreover, the decline in phytoplasma titer observed in CLI and leaves showing WB, later phases of infection compared to VIR, and PHY/BB (Figure 5 and Figure 6) reflects a strategic balance for mutual survival. Since phytoplasmas are exclusively intracellular bacteria, their survival is linked to the host plant’s health. Reduced pathogen load in late-stage infections is a compromise to prevent overwhelming the host and excessive damage to the plant by phytoplasmas. This dynamic indicates the complex interactions between pathogen virulence and host viability.

In addition, in the direct measurement of PPT titer and the relative ratio of PPT to plant DNA by qPCR, a distinctive pattern was observed; that is, periwinkle plants harbored a substantially higher PPT phytoplasma load in comparison to tomato plants (PHY and BB, Figure 5 and Figure 6). This discrepancy in the phytoplasma concentration highlights the distinct susceptibility of periwinkle and tomato to PPT phytoplasma infection. Periwinkles might provide a more conducive environment for phytoplasma growth or may have less effective defense responses compared to tomatoes. The variations in host responses to phytoplasma infection may be attributed to differences in physiology, genetics, and defense mechanisms of these two plant species. The variation in pathogen load across different plant hosts is a well-established phenomenon [35,36,37,38,39]; for instance, the population sizes of *Xanthomonas campestris* pv. *vitians* change in lettuce with different genotypes [36]. Another example is the differing susceptibility of grapevine varieties to *Plasmopara viticola*, the causal agent of downy mildew, where specific grapevine cultivars support higher pathogen loads due to genetic differences in disease resistance [35].

This study provided insights into the dynamic variations of PPT phytoplasma concentrations in periwinkle and tomato, shedding light on the host-specific responses to phytoplasma infection. The combined utilization of qPCR and western blot analysis has facilitated the assessment of phytoplasma titer, enhancing the accuracy and reliability of our results. The observed disparities in phytoplasma titer and symptom development between periwinkle and tomato highlight the intricacies of host-pathogen interactions and underline the necessity for further research to unravel the underlying mechanisms governing these variations. Understanding these interactions holds importance for developing effective strategies to manage phytoplasma infections in diverse plant species, ultimately benefiting agricultural and horticultural practices.

## 4. Materials and Methods

### 4.1. Potato Purple Top (PPT) Phytoplasma

The Columbia Basin PPT phytoplasma, also referred to as beet leafhopper-transmitted virescence agent (BLTVA), was detected in potatoes exhibiting disease symptoms in potato production fields situated in Washington and Oregon [40,41]. The PPT phytoplasma strain was maintained using its alternate hosts, namely tomato (*Solanum lycopersicum* cv. Moneymaker) and Madagascar periwinkle (*Catharanthus roseus* (L.) Vinca Pacifica XP pure white), within a controlled greenhouse environment.

### 4.2. Graft Inoculation

Phytoplasma infection in tomato plants was established by employing small seedling grafting inoculation developed earlier [12]. Healthy tomato seedlings at the four-leaf stage (<one month old) were chosen as rootstocks, and shoots displaying WB symptoms from infected plants served as phytoplasma inocula (scions). To prepare the inoculum, the lower end of a freshly cut shoot was trimmed into a wedge, which was then inserted into a cleft cut in the main stem of the recipient plant. A plastic grafting clip was employed to tighten the graft union securely. Subsequently, these grafted seedlings were placed in oversized domed propagators (EarlyGrow, Pickering, North Yorkshire, UK) for two weeks to maintain the optimal moisture level. A similar approach, through grafting, was employed to establish PPT phytoplasma infection in periwinkle plants. The only difference was the use of 3-month-old young periwinkle seedlings for grafting. All grafted plants, whether tomato or periwinkle, were maintained in a greenhouse with a photoperiod of 16 h of light followed by 8 h of darkness. The day of graft inoculation was designated as day 0, representing the moment when the host plant was initially exposed to the phytoplasma inoculum.

### 4.3. Symptom Observation and Stereomicroscopic Imaging

Following the graft inoculation with PPT phytoplasma, daily monitoring and recording of disease symptom development was conducted in the infected plants. The dissected flower buds from mock-inoculated and infected plants were examined under a stereomicroscope (Stereo Discovery V20; Zeiss, Jena, Germany). The images were captured using a digital camera (AxioCam; Zeiss, Jena, Germany) attached to the microscope for further analysis.

### 4.4. Sample Collection and DNA Extraction

A total of three biological samples were collected for each distinct symptom that manifested in tomato and periwinkle plants infected with PPT phytoplasma. In tomato plants, these symptoms included BB, CLI, and WB (Wei et al., 2013; this study). In periwinkle plants, the symptoms included VIR, PHY, and WB (See the Result Section). To extract plant and phytoplasma total DNA, the DNeasy Plant Mini kit (Qiagen, Valencia, CA, USA) was employed according to the manufacturer’s instructions.

### 4.5. PCR Amplification and Cloning of Marker Genes from PPT Phytoplasma, Tomato, and Periwinkle

The PPT phytoplasma 16S rRNA gene was amplified by semi-nested PCR using universal primer pairs, specifically P1/16S-SR [24,25] and P1A/16S-SR [25], as listed in Table 2. In addition, primer pairs Tom18SbF1/Tom18SbR1 and CrAct3bF1/CrAct3bR1 (Table 2) were designed by utilizing the Primer3Plus online tool [42]. These two primers target the fragments of the 18S rRNA gene (OK073663.1) and the actin gene (MG813871.1) specific to tomato and periwinkle plants, respectively. These two genes were amplified by PCR.

PCR reactions were conducted in 25 µL reaction mixtures, comprising 1 µL of undiluted total DNA, 200 µM of each dNTP, 0.4 µM of each primer, 15 mM MgCl_2_, and 2.5 units of LA Taq DNA polymerase (Takara Bio Madison, WI, USA). The PCR amplification followed the thermocycling conditions: initial denaturation at 94 °C for 1 min, annealing at 55 °C for 2 min, and primer extension at 72 °C for 3 min (with a final extension of 10 min). For semi-nested PCR, 1 µL of a 1:20 diluted PCR product from the first amplification served as the template, using similar thermocycling conditions. These conditions were also employed to amplify 18S rRNA and actin gene fragments, with the only variation being an annealing temperature of 57 °C.

Subsequently, the PCR products were subjected to electrophoresis on a 1% agarose gel containing SYBR Safe DNA Gel Stain (Invitrogen, Carlsbad, CA, USA) and visualized using a UV transilluminator. The PCR amplicons for the 16S rRNA, 18S rRNA, and actin genes were cloned into a TOPO TA cloning kit (Invitrogen, Carlsbad, CA, USA) and transformed to TOP10 Electrocomp™ Cells (Invitrogen, Carlsbad, CA, USA). Recombinant plasmids were then purified using the Bio-Rad Quantum Prep Plasmid Miniprep kit (Bio-Rad, Hercules, CA, USA).

### 4.6. Standard Curve Generation and qPCR Analysis

Plasmid DNAs containing the 16S rRNA gene of the PPT phytoplasma, as well as fragments of the tomato 18S rRNA and periwinkle actin genes, were quantified using a NanoDrop One Microvolume UV-Vis Spectrophotometer (Thermo Scientific, Madison, WI, USA). Subsequently, these plasmids with known concentrations underwent a tenfold serial dilution, and qPCR was performed by AriaMX qPCR system (Agilent Technologies, Santa Clara, CA, USA) to determine the Ct values. The primer pairs MPPLPPT16SF2/MPPLPPT16SR2 [21], Tom18SinF1/Tom18SbR1, and CrAct3inF2/CrAct3bR2 (Table 2) were employed to amplify the PPT phytoplasma 16S rRNA, tomato 18S rRNA and periwinkle actin genes, respectively. The standard curves were then generated by plotting the logarithm of the known DNA concentrations against their corresponding Ct values, following the methodology outlined by Jawhari et al. (2015, [31]). The copy numbers of plasmid DNA molecules in each dilution were calculated using the DNA copy number calculator (https://horizondiscovery.com/en/ordering-and-calculation-tools/dna-copy-number-calculation, accessed on 18 October 2023). These standard curves served as references for quantifying PPT phytoplasma and plant DNA in the samples collected from different symptom stages based on their Ct values. Statistical analysis was conducted using the Student’s *t*-test within Microsoft Excel Version 2401 (Microsoft Corporation, Seattle, WA, USA) to ascertain the significance of the distinctions among the groups.

For the qPCR reactions, each contained 10 µL of Brilliant III Ultra-Fast SYBR Green Low ROX qPCR Master Mix (Agilent Technologies, Santa Clara, CA, USA), 2 µL of sample DNA, 0.3 µM of each primer, and 5.6 µL of water. The qPCR thermal profile included an initial step at 95 °C for 3 min, followed by 40 cycles of denaturation at 95 °C for 5 s and annealing at 57 °C for 10 s. Data acquisition and analysis were performed using Aria software version 1.6 (Agilent Technologies, Santa Clara, CA, USA).

### 4.7. Western Blot Analysis

The western blot analysis utilized the polyclonal anti-PPT-IDP antibody, specifically targeting the immunodominant protein of PPT phytoplasma. This antibody was produced in rabbits in our previous study [8]. First, proteins were extracted from distinct symptomatic tissues (100 mg) representing various infection stages of PPT phytoplasma-infected tomato and periwinkle plants, along with corresponding mock controls. The extraction was carried out using a method previously described by Inaba and Nagy in 2018 [43]. The proteins were separated on a Novex™ WedgeWell™ 4–20% Tris-Glycine gel (Invitrogen, Waltham, MA, USA). Subsequently, the gel was transferred onto a PVDF membrane using Tris-Glycine Transfer buffer (25 mM Tris, 192 mM glycine) at 400 mA for 90 min. The transferred membrane was blocked with TBS-T (50 mM Tris-HCl, pH 7.5, 150 mM NaCl, 0.1% Tween-20) containing 5% non-fat dry milk and then reacted with the anti-PPT-IDP at 4 °C overnight. The target proteins were detected using an anti-rabbit secondary antibody conjugated with alkaline phosphatase (Promega, Madison, WI, USA) and visualized by a colorimetric method. The intensity of bands was evaluated using Adobe Photoshop software’s histogram function (https://helpx.adobe.com/photoshop/using/viewing-histograms-pixel-values.html) (accessed on 27 December 2023).

## Figures and Tables

**Table 1:** Comparison of the sepals (the outermost whorl of flower organs) between mock-inoculated flowers and big buds (BBs) induced by infection with potato purple top (PPT) phytoplasma in tomato plants.

Plant Sample		Shape	Weight (Gram)	Length (Centimeter)	Width (Centimeter)
mock-inoculated	Sepals-flower1	Separated	0.018	1.6	0.7
	Sepals-flower2	Separated	0.016	1.2	0.5
	Sepals-flower3	Separated	0.028	1.7	0.6
	Sepals-flower4	Separated	0.018	1.5	0.6
	Mean ± SD	NA	0.021 ± 0.01	1.467 ± 0.21	0.6 ± 0.08
PPT-infected	Sepal-BB1	fused	0.217	5	3.2
	Sepal-BB2	fused	0.165	4.5	2.5
	Sepal-BB3	fused	0.223	4.5	2.4
	Sepal-BB4	fused	0.222	3.5	2.6
	Mean ± SD	NA	0.207 ± 0.02	4.375 ± 0.54	2.675 ± 0.31

SD: standard deviation. NA: not available.

**Table 2:** The primers used in this study for PCR amplification and quantitative PCR (qPCR) analysis of target genes, including the 16S rRNA gene of potato purple top (PPT) phytoplasma, 18S rRNA gene of tomato, and the periwinkle actin gene.

Target Gene		Primer Name	Primer Sequence	Reference
PCR amplification of target genes	16S rRNA (PPT)	P1	AAGAGTTTGATCCTGGCTCA	[24]
		P1A	AACGCTGGCGGCGCGCCTAATAC	[25]
		16S-SR	GGTCTGTCAAAACTGAAGATG	[25]
	18S rRNA (tomato)	Tom18SbF1	ATTGGAGGGCAAGTCTGGTG	This study
		Tom18SbR1	GCGATCCGAACATTTCACCG	This study
	Actin (periwinkle)	CrAct3bF1	TTGTTGGTCGCCCTAGACAC	This study
		CrAct3bR1	GTGATGCCAAGATGGAGCCT	This study
qPCR analysis of target genes	16S rRNA (PPT)	MPPLPPT16SF2	AGGGTGCGTAGGCTGTTAGA	[21]
		MPPLPPT16SR2	TGCCTCAGCGTCAGTAAAGA	[21]
	18S rRNA (tomato)	Tom18SinF1	ACAGGCCCGGGTAATCTTTG	This study
		Tom18SbR1	GCGATCCGAACATTTCACCG	This study
	Actin (periwinkle)	CrAct3inF2	CGGCAACATTGTACTCAGTGG	This study
		CrAct3bR2	TGCTCATCCTATCGGCGATG	This study

## Data Availability

The data presented in this study are available in the article text.
